# The space of phylogenetic mixtures for equivariant models

**DOI:** 10.1186/1748-7188-7-33

**Published:** 2012-11-28

**Authors:** Marta Casanellas, Jesús Fernández-Sánchez, Anna M Kedzierska

**Affiliations:** 1 Departament de Matemàtica Aplicada I, ETSEIB, Universitat Politècnica de Catalunya, Avinguda Diagonal 647, Barcelona, 08028, Spain; 2 Centre for Genomic Regulation (CRG), Dr. Aiguader 88, Barcelona, 08003, Spain

**Keywords:** Evolutionary model, Equivariant model, Phylogenetic mixture, Identifiability

## Abstract

**Background:**

The selection of an evolutionary model to best fit given molecular data is usually a heuristic choice. In his seminal book, J. Felsenstein suggested that certain linear equations satisfied by the expected probabilities of patterns observed at the leaves of a phylogenetic tree could be used for model selection. It remained an open question, however, whether these equations were sufficient to fully characterize the evolutionary model under consideration.

**Results:**

Here we prove that, for most equivariant models of evolution, the space of distributions satisfying these linear equations coincides with the space of distributions arising from mixtures of trees. In other words, we prove that the evolution of an observed multiple sequence alignment can be modeled by a mixture of phylogenetic trees under an equivariant evolutionary model if and only if the distribution of patterns at its columns satisfies the linear equations mentioned above. Moreover, we provide a set of linearly independent equations defining this space of phylogenetic mixtures for each equivariant model and for any number of taxa. Lastly, we use these results to perform a study of identifiability of phylogenetic mixtures.

**Conclusions:**

The space of phylogenetic mixtures under equivariant models is a linear space that fully characterizes the evolutionary model. We provide an explicit algorithm to obtain the equations defining these spaces for a number of models and taxa. Its implementation has proved to be a powerful tool for model selection.

## Background

The principal goal of phylogenetics is to reconstruct the ancestral relationships among organisms. Most popular phylogenetic reconstruction methods are based on mathematical models describing the molecular evolution of DNA. In spite of this, there exists no unified framework for model selection and the results are highly dependent on the models and methods used in the analysis (cf. [1]).

In this paper we assume the Darwinian model of evolution proceeding along phylogenetic trees and address the following question: how can the data evolving under a particular model be characterized? In other words, we look for invariants of the DNA patterns which have evolved following a tree (or a mixture of trees, as we will see below) under a particular model. The answer to this question provided in this paper leads to a complete characterization of the evolutionary model and to a novel model selection tool, which is valid for any mixture of trees.

In what follows, we briefly explain the motivation for this work. It has been shown that if the evolution along a phylogenetic tree is described by a particular model, the expected probabilities of nucleotide patterns at the leaves of the tree satisfy certain equalities (see e.g. [2], p.375). Several authors (e.g. [2-4]) pointed out that these equalities could potentially be used to test the fitness of the model of base change. The full set of equations required for viable model selection, however, was unknown. The objective of this work is to fill in this gap and to go a step further into practical aplication by providing an algorithm to compute the required invariants for model selection.

In this work we consider a group of equivariant models ([5,6]). These models are Markov processes on trees, whose transition matrices satisfy certain symmetries: the Jukes-Cantor model, the Kimura 2 and 3 parameter models, the strand symmetric model, and the general Markov model. Our first important result, Theorem 17, states that if evolution occurs according to trees (or even mixtures of trees) under these equivariant models, then the model of evolution is completely determined by the linear space defined by the aforementioned equalities. By exhaustively studying the group of symmetries of these models, we also give a straightforward combinatorial way of determining the equations of this linear space (see Theorem 22). The implementation of the algorithm producing the equations is available as a package SPIn ([7], http://genome.crg.es/cgi-bin/phylo_mod_sel/AlgModelSelection.pl.), which has proved to be a successful tool in evolutionary model selection.

Our main technique consists in proving that the linear space above coincides with the space $D^{M}$ of *phylogenetic mixtures* evolving under the model , i.e. the set of points that are linear combinations of points lying in the phylogenetic varieties $CV_{T}^{M}$ (see Preliminaries section for specific definitions). In biological words and in the stochastic context, this is the set of vectors of expected pattern frequencies for mixtures of trees evolving under the model  (not necessarily the same tree topology in the mixture, and not necessarily the same transition matrices when the tree topologies coincide). In phylogenetics, the so-called i.i.d. hypothesis (independent and identically distributed) about the sites of an alignment is prevalent in the most simple models. When the assumption “identically distributed” is replaced it by “distributed according to the same evolutionary model”, one obtains a phylogenetic mixture.

Phylogenetic mixtures are useful in modeling heterogeneous evolutionary processes, e.g. data comprising multiple genes, selected codon positions, or rate variation across sites (e.g. [8]). Among a plethora of applications, they are used in orthology predictions, gene and genome annotations, species tree reconstructions, and drug target identifications.

In addition to the main result, we determine the dimension of these linear spaces and use it to give an upper bound, *h*_0_(*n*), on the number of mixtures that should be used in phylogenetic reconstruction on *n* taxa. This relates to the so-called *identifiability* problem in phylogenetic mixtures, which can be posed as determining the conditions that guarantee that the model parameters (discrete parameters in the form of tree topologies and the continuous parameters of the root and model distributions) can be recovered from the data. Identifiability is crucial for consistency of the maximum likelihood approaches and, though extensively studied in the phylogenetic context, few results are known (see for instance [9-13]).

In brief, in Theorem 30 we prove that either the tree topologies or the continuous parameters are not generically identifiable for mixtures on more than *h*_0_(*n*) trees under equivariant models. Here *h*_0_(*n*) is the quotient of the dimension of the linear space $D^{M}$ (computed in Proposition 20) by the number of free parameters of  on a trivalent tree plus one. For example, for four taxa and the Jukes-Cantor model (resp. the Kimura 3-parameter model) this result proves that mixtures on three (resp. four) or more taxa are not identifiable (i.e. either the discrete or the continuous parameters cannot be fully identified). A detailed discussion on this subject is provided in the last section.

The main tools used in this work are algebraic geometry and group theory. The reader is referred to [14,15] for general references on these topics.

## Main text

### Preliminaries

Phylogenetic trees and Markov models of evolution have been widely used in the literature. In what follows we fix the notation needed to deal with them in our setting.

Let *n* be a positive integer and denote by [*n*] the set {1,2,…,*n*}. A *phylogenetic treeT* on the set of taxa [*n*] is a tree (i.e. a connected graph with no loops), whose *n* leaves are bijectively labeled by [*n*]. Its vertices represent species or other biological entities and its edges represent evolutionary processes between the vertices.

We allow internal vertices of any degree and if all the internal vertices are of degree 3 we say that the tree is *trivalent*. We will denote the set of vertices of *T* by *N*(*T*), the set of edges by *E*(*T*), and the set of interior nodes by *Int*(*T*). A *rooted tree* is a tree together with a distinguished node *r* called the *root*. The root induces an orientation on the edges of *T*, whereby the root represents the common ancestor to all the species represented in the tree. If *e* is an edge of a rooted tree *T*, we write *pa*(*e*) and *ch*(*e*) for its parent vertex (origin) and its child vertex (end), respectively. Two unrooted phylogenetic trees on the set of taxa [*n*] are said to have the *same tree topology* if their labeled graphs have the same topology.

We fix a positive integer *k* and an ordered set *B* = {*b*_1_,*b*_2_,…,*b*_*k*_}. For example, for most applications we take *B* = {A,C,G,T} to be the set of nucleotides in a DNA sequence. We may think of *B* as the set of states of a discrete random variable. We call *W* the complex vector space $W={\langle B\rangle }_{C}$ spanned by *B*, so that *B* is a natural basis of *W*. For algebraic convenience, we usually work over the complex field and restrict to the stochastic setting when necessary. Vectors in *W* are thought of as probability distributions on the set of states *B* if their coordinates are non-negative and sum to one. In this setting the vector $\sum c_{i}b_{i}$ means that observation *b*_*i*_ occurs with probability *c*_*i*_. From now on, we will identify vectors in *W* with their coordinates in the basis *B* written as a column vector, e.g. we identify $\sum_{k}b_{k}$ with the vector **1 **= (1,1,…,1)^*t *^∈* W*.

In order to model molecular evolution on a phylogenetic tree *T*, we consider a Markov process specified by a root distribution, *Π*∈*W*, and a collection of transition matrices, **A **= (*A*^*e*^)_*e*∈*E*(*T*)_, where each *A*^*e*^is a *k *×* k*-matrix in *End*(*W*). The matrices *A*^*e*^represent the conditional probabilities of substitution between the states in *B* from the parent node *pa*(*e*) to the child node *ch*(*e*) of *e*. We adopt the convention that the matrices *A*^*e*^ act on *W* from the right, i.e. a vector *ω*^*t *^in *pa*(*e*) maps to *ω*^*t*^*A*^*e*^ in *ch*(*e*).

Distinct forms of the transition matrices give rise to different evolutionary models. Using the terminology introduced above, we proceed to the definition of evolutionary models used throughout this work.

**Definition 1.** An *(algebraic) evolutionary model* is specified by giving a vector subspace $W_{0}\subset W$ such that **1**^*t *^*Π *≠ 0 for some *Π *in *W*_0_, together with a multiplicatively closed vector subspace *Mod*(for *model*) of $M_{k}(C)$ containing the identity matrix. We will usually denote such a model by $M=(W_{0},\text{Mod})$. We define the *stochastic evolutionary model*$sM=(sW_{0},\text{sMod})$*associated to* by taking *sW*_0 _= {*Π*∈*W*_0_:**1**^*t *^*Π *= 1} and *sMod *= {*A*∈*Mod*:*A***1 **=** 1**}. The term “stochastic” refers to the fact that, by restricting to the points in the spaces with non-negative real entries, we obtain distributions and Markov matrices. A phylogenetic tree *T* together with the parameters *Π*and **A **= (*A*^*e*^) _*e *∈* E*(*T*)_is said to *evolve under the algebraic evolutionary model* if *Π *∈* W*_0_, and all matrices *A*^*e *^lie in *Mod*.

**Remark 2.** Note that *sW*_0_and *sMod* are not vector spaces. The condition **1**^*t *^*Π *≠ 0 in the above definition means that the sum of the coordinates of *Π *is not zero. Since vectors in *sW*_0 _with non-negative coordinates represent the probability distributions for the set of observations *B*, this condition implies no restriction from a biological point of view. Moreover, it ensures that $W_{0}\cap \{\sum_{x\in B}\Pi_{x}=1\}$ has dimension equal to *dim*(*W*_0_) − 1. In particular, the simplex of stochastic vectors in *W*_0_will form a semialgebraic set of ${\langle B\rangle }_{R}$ of dimension equal to *dim*(*W*_0_)−1 (as expected).

**Remark 3.** The subspace *Mod* of substitution matrices is usually required to be multiplicatively closed (as in the definition above) so that when two evolutionary processes are concatenated, the final process is of the same kind. The importance of this requirement is the starting point of [16], where a different approach to the definition of “evolutionary mode” is provided.

Our definition of evolutionary models includes most of the well-known evolutionary models, namely those given in [17] and the *equivariant* models (see [5,6]).

**Example 4.** Let *G* be a permutation group of *B*, that is, a group whose elements are permutations of the set *B*, $G\leq S_{k}$. Given *g *∈* G*, write *P*_*g*_ for the *k *×* k*-permutation matrix corresponding to *g*: (*P*_*g*_)_*i*,*j *_= 1 if *g*(*j*) =* i* and 0 otherwise. The *G*-*equivariant evolutionary model*$M_{G}$ is defined by taking *Mod* equal to

$$\mathrm{m:m}(G)=\{A\in {\mathrm{m:m}}_{k}(C)|P_{g}AP_{g}^{-1}=A\;\text{for all}\;g\in G\},$$

and *W*_0 _= {*Π *∈* W *∣* P*_*g *_*Π *=* Π *for all* g *∈* G*}. These subsets are vector subspaces of $M_{k}(C)$ and *W*, respectively. Moreover, if *A*_1_,*A*_2 _∈* M*(*G*), then

$$P_{g}A_{1}A_{2}P_{g}^{-1}=(P_{g}A_{1}P_{g}^{-1})(P_{g}A_{2}P_{g}^{-1})=A_{1}A_{2},$$

and *A*_1_*A*_2 _∈* M*(*G*). Therefore, equivariant models provide a wide family of examples of algebraic evolutionary models in the sense of Definition 1. For example, if *B *= {A,GT}, it can be seen that the algebraic versions of the Jukes-Cantor model [18], the Kimura models with 2 or 3 parameters [19,20], the strand symmetric model [21] or the general Markov model [22] are instances of equivariant models: 

• if $G=S_{4}$, then $M_{G}$ is the *algebraic Jukes-Cantor model*JC69,

• if *G *= 〈(ACGT),(AG)〉, then $M_{G}$ is the *algebraic Kimura 2-parameter model*K80,

• if *G *= 〈(AC)(GT),(AG)(CT)〉, then $M_{G}$ is the *algebraic Kimura 3-parameter model*K81,

• if *G *= 〈(AT)(CG)〉, then $M_{G}$ is known as the *strand symmetric model*SSM, and

• if *G *= 〈*e*〉, then $M_{G}$ is the *general Markov model*GMM.

Given an evolutionary model  and a phylogenetic tree *T*, we define the *space of parameters* as

$${\text{Par}}_{M}\left(T\right)=W_{0}\times \left(\underset{e\in E(T)}{\prod }\text{Mod}\right).$$

Similarly, we define the space of *stochastic* parameters associated to *T* by

$${\text{Par}}_{sM}\left(T\right)=sW_{0}\times \left(\underset{e\in E(T)}{\prod }\text{sMod}\right).$$

Though artificial at first glance, the use of tensors in the framework that includes the distributions on the set of patterns in *B* at the leaves of a phylogenetic tree is a natural choice. Indeed, if $p_{x_{1}x_{2}\ldots x_{n}}$ denotes the joint probability of observing x_1 _at leaf 1, x_2_ at leaf 2, and so on, up to x_*n*_ at leaf *n*, then the vector $p=(p_{b_{1}\ldots b_{1}},p_{b_{1}b_{1}\ldots b_{2}},\ldots ,p_{b_{k}\ldots b_{k}})$ provides a distribution on the set of patterns in *B* at the leaves of *T*, and this can be regarded as the tensor having these coordinates in the natural basis,

$$p=\underset{x_{1}\ldots x_{n}\in B}{\sum }p_{x_{1}\ldots x_{n}}x_{1}⊗\ldots ⊗x_{n}\text{.}$$

This motivates the following definition.

**Definition 5.** Given a phylogenetic tree *T* on the set of taxa [*n*], an [*n*]-*tensor* is any element of the tensor power

$$L:={⊗}_{\left[n\right]}\mathrm{W.}$$

Given an algebraic evolutionary model  and a phylogenetic tree *T* with root *r*, every Markov process on *T* (specified by a collection of parameters *Π* and **A **= (*A*_*e*_)_*e*∈*E*(*T*)_) gives rise to a tensor in  in the following way: we consider a *parametrization*

(1) $$\Psi_{T}^{M}:{\text{Par}}_{M}(T)\to L$$

defined by

$$\Psi_{T}^{M}\left(\Pi ,A\right)=\underset{x_{i}\in B}{\sum }p_{x_{1}\ldots x_{n}}x_{1}⊗\cdots ⊗x_{n},$$

where

(2) $$p_{x_{1}\ldots x_{n}}=\underset{x_{v}\in B,v\in \mathrm{Int}(T)}{\sum }\Pi_{x_{r}}\underset{e\in E(T)}{\prod }A_{x_{\text{pa(e)}},x_{\text{ch(e)}}}^{e},$$

x_*v*_ denotes the state at the vertex *v*, *pa*(*e*) (resp. *ch*(*e*)) is the parent (resp. child) node of *e*, and *Π*_x_,x ∈* B*, are the coordinates of *Π*. When restricted to the stochastic matrices and distributions in *W*_0_, this parametrization corresponds to the hidden Markov process on the tree *T* (the leaves correspond to the observed random variables and the interior nodes to the hidden variables).

The parametrization (1) restricts to another polynomial map $\phi_{T}^{M}:{\text{Par}}_{sM}(T)\to H$, where H⊂L is the hyperplane defined by $H=\left\{p\in L|\sum_{x_{1},\ldots ,x_{n}\in B}p_{x_{1}\ldots x_{n}}=1\right\}$. Because we work in the algebraic setting, the use of the word “stochastic” in this paper is more general than usual, as we only request entries summing to one.

From now on, we will refer to this restriction as the *stochastic parametrization*$\phi_{T}^{M}$. It is important to note that when we consider the distributions in *sW*_0_ and the Markov matrices in *sMod*, its image by $\phi_{T}^{M}$ lies in the standard simplex in  (and thus in *H*). This in turn implies that the whole image $\text{Im}\;\phi_{T}^{M}$ is contained in *H*.

We proceed to define the algebraic varieties associated to the parametrization maps defined above. Roughly speaking, algebraic varieties are sets of solutions to systems of polynomial equations (e.g. [14]).

**Definition 6.** The *stochastic phylogenetic variety*$V_{T}^{M}$*associated to a phylogenetic treeT* is the smallest algebraic variety containing $\text{Im}\;\phi_{T}^{M}=\left\{\phi_{T}^{M}(\Pi_{r},A):\right)\left(\left(\Pi_{r},A)\in {\text{Par}}_{sM}(T\right)\right\}$ (in particular, $V_{T}^{M}\subset H$).

Similarly, the *phylogenetic variety*$CV_{T}^{M}$*associated toT* is the smallest algebraic variety in  that contains $\text{Im}\;\Psi_{T}^{M}=\left\{\Psi_{T}^{M}(\Pi_{r},A):(\Pi_{r},A)\in {\text{Par}}_{M}(T)\right\}$.

Below we explain the reason for the notation of $CV_{T}^{M}$, which was adopted from [23].

**Figure 1:**
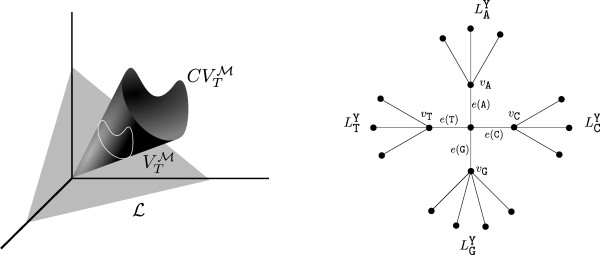
**On the left, the varieties**$V_{T}^{M}$**and**${\mathrm{CV}}_{T}^{M}$**are shown; on the right, the phylogenetic tree described in the proof of Proposition 13 is represented.**

The reader may note that the position of the root *r* of *T* played a role in the above parameterizations. It can be shown, however, that under certain mild assumptions, $\text{Im}\;\Psi_{T}^{M}$ and $\text{Im}\;\phi_{T}^{M}$ are independent of the root position in the following sense: if two phylogenetic trees have the same topology as unrooted trees, then the smallest algebraic varieties containing the corresponding image sets are the same. For example, any model $M=(W_{0},\text{Mod})$ satisfying (i) ${\tilde{\Pi }}^{t}:=\Pi^{t}A$ belongs to *W*_0_ for all *Π *∈* W*_0_ and all *A *∈* Mod*, and (ii) $D_{\tilde{\Pi }}^{-1}A^{t}D_{\Pi }\in \text{Mod}$ whenever $D_{\tilde{\Pi }}^{-1}$ exists (here *D*_*ω*_ denotes the diagonal matrix with the entries of *ω *on the diagonal and zeros elsewhere) has this property (in this case, we say the model is *root-independent*). It is not difficult to check that the equivariant models satisfy these two properties (e.g. adapting the proof of [24] or [25]). For technical reasons, from now on we consider only the evolutionary models satisfying (i) and (ii). Indeed, in this case the notation $CV_{T}^{M}$ refers to the fact that the phylogenetic variety is just the cone over the stochastic phylogenetic variety (see Figure 1 and the remark below).

**Remark 7.** Let  be an evolutionary model satisfying (i) and (ii) above. For p∈L, $p=\sum p_{x_{1}\ldots x_{n}}x_{1}⊗\ldots ⊗x_{n}$, define $\lambda (p):=\sum_{x_{i}\in B}p_{x_{1}\ldots x_{n}}$. Then

$$CV_{T}^{M}=\left\{p\in L|\;p=\lambda (p)q\;,q\in V_{T}^{M}\right\}$$

and $V_{T}^{M}=CV_{T}^{M}\cap H$. This is well known for the general Markov model [23] and can be easily generalized to any model satisfying (i) and (ii).

### The space of phylogenetic mixtures

In phylogenetics, the hypothesis that the sites of an alignment are independent and identically distributed is often used. When the assumption “identically distributed” is replaced by “distributed according to the same evolutionary model”, one obtains a phylogenetic mixture. Below, we introduce phylogenetic mixtures from the algebraic point of view (see also [26]).

**Definition 8.** Fix a set of taxa [*n*] and an algebraic evolutionary model . A *phylogenetic mixture (on m-classes)* or *m-mixture* is any vector $p\in L={⊗}_{\left[n\right]}W$ of the form

$$p=\overset{m}{\underset{i=1}{\sum }}\alpha_{i}p^{i},$$

where $\alpha_{i}\in C$ and $p^{i}\in \text{Im}(\Psi_{T_{i}}^{M})$ for some tree topologies *T*_*i*_ on the set of taxa [*n*]. As $\Psi_{T_{i}}^{M}$ is a homogeneous map, phylogenetic mixtures are represented by vectors of the form $\sum_{i=1}^{m}{\overset{ˇ}{p}}^{i}$, where ${\overset{ˇ}{p}}^{i}\in \text{Im}(\Psi_{T_{i}}^{M})$. We call $D_{M}\subset L$ the *space of all phylogenetic mixtures* (on any number of classes) under the algebraic evolutionary model .

As mentioned in the introduction, the tree topologies contained in the mixture can be the same or different. An example of a phylogenetic mixture is the data modeled by the discrete Gamma-rates models (see e.g. [8]).

Restricting matrix rows to sum to one requires restricting the phylogenetic mixtures to the points of the form

$$q=\overset{m}{\underset{i=1}{\sum }}\alpha_{i}q^{i}\;\text{where}\;q^{i}\in \text{Im}(\phi_{T_{i}}^{M})\;,\;\text{and}\;\sum_{i}\alpha_{i}=1.$$

We call $D_{sM}$ the space of *stochastic phylogenetic mixtures*.

**Remark 9.** The phylogenetic variety of a trivalent tree topology contains all phylogenetic varieties of the non-trivalent tree topologies obtained by contracting any of its interior edges. Indeed, the latter are a particular case of the former when the matrices associated to the contracted edges are equal to the identity matrix. It follows that the space of phylogenetic mixtures on the trivalent tree topologies coincides with the space of phylogenetic mixtures on all possible topologies.

The following result was proven by Matsen, Mossel and Steel in [26] for the two state random cluster model but, as proved below, it can be easily generalized to any evolutionary model.

**Lemma 10.** Given a set of taxa [*n*] and an algebraic evolutionary model , the set of all phylogenetic mixtures $D_{M}$ is a vector subspace of . Similarly,  is a linear variety and it equals $D_{M}\cap H$.

*Proof.*$D_{M}$ is a -vector space and $D_{sM}$ is a linear variety by their definition. It follows that $D_{M}$ is an algebraic variety that contains $\text{Im}\;\Psi_{T}^{M}$ for any phylogenetic tree *T* on the set of taxa [*n*]. Therefore, it also contains $CV_{T}^{M}$, and $D_{M}$ equals the set of points of the form $p=\sum p_{i}$, where $p_{i}\in CV_{T_{i}}^{M}$. Similarly, $D_{sM}$ is an algebraic variety that contains $\text{Im}\;\phi_{T}^{M}$, so it also contains $V_{T}^{M}$ for any phylogenetic tree *T*. It follows that $D_{sM}$ is formed by points of type $q=\sum \alpha_{i}q_{i}$, where $q_{i}\in V_{T_{i}}^{M}$ and $\sum_{i}\alpha_{i}=1$.

Now we check that $D_{sM}=D_{M}\cap H$. Let $q\in D_{sM}$, so that $q=\sum_{i=1}^{m}\alpha_{i}q^{i}$ for some *m*, $q^{i}\in V_{T_{i}}^{M}$, and $\sum \alpha_{i}=1$. Clearly, $q\in D_{M}$. Moreover, the sum of coordinates of *q*, *λ*(*q*), satisfies $\lambda (q)=\sum_{i}\alpha_{i}\lambda (q^{i})=\sum_{i}\alpha_{i}=1$. Thus, *q *∈* H*. Conversely, let $p=\sum_{i=1}^{m}p^{i}$ with $p^{i}\in CV_{T_{i}}^{M}$ for certain tree topologies *T*_*i*_, and assume that *λ*(*p*) = 1. Apply Remark 7 to each *p*^*i *^to get *p*^*i *^=* λ*(*p*^*i*^)*q*_*i*_ for some $q_{i}\in V_{T_{i}}^{M}$. Then

$$p=\sum_{i}p^{i}=\sum_{i}\lambda (p^{i})q_{i}$$

and $1=\lambda (p)=\sum_{i}\lambda (p^{i})\lambda (q_{i})=\sum_{i}\lambda (p^{i})$ since each *q*_*i*_ lies on *H*. This proves that $p\in D_{sM}$. □

**Remark 11.** In the proof of the above lemma, we have seen that $D_{M}$ and $D_{sM}$ can be alternatively described as the spaces of mixtures obtained from the respective varieties $CV_{T}^{M}$ and $V_{T}^{M}$ (i.e., not only from the images of the parametrization maps).

### The space of phylogenetic mixtures for equivariant evolutionary models

This section provides a precise description of the space $D_{M}$ for the equivariant models  listed in Example 4 (JC69, K80, K81, SSM, and GMM). First, we recall some definitions and facts of group theory and linear representation theory. From now on, *B *= {A,C,G,T}, *k *= 4, $W={\langle B\rangle }_{C}$, *n* is fixed and $L={⊗}_{\left[n\right]}W$.

#### Background on representation theory

We introduce some tools in group representation theory needed in the sequel. We refer the reader to [15] as a classical reference for these concepts. Although some of the following results are valid for any permutation group, for simplicity in the exposition we restrict to permutations of four elements (as our applications deal only with the case *B *= {ACGT}).

Let $G\leq S_{4}$ be a permutation group. The trivial element in $S_{4}$ will be denoted as *e*. We write *ρ*_*G*_ for the restriction to *G* of the *defining* representation $\rho :S_{4}\to \mathrm{GL}(W)$ given by the permutations of the basis *B* of *W*. This representation induces a *G*-module structure on *W* by setting *g *· x: =* ρ*(*g*)(x)∈* W*. In fact, *ρ* induces a *G*-module structure on any tensor power ⊗^*s*^*W*by setting

(3) $$g\cdot \left(x_{1}⊗\ldots ⊗x_{s}\right):=g\cdot x_{1}⊗\ldots ⊗g\cdot x_{s},$$

and extending by linearity. From now on, the space $L={⊗}^{n}W$ will be implicitly considered as a *G*-module with this action. We call *χ* the character associated to the representation *ρ*_*G*_:*G *→* GL*(*W*), i.e. *χ*(*g*) is the trace of the corresponding permutation matrix or, in other words, *χ*(*g*) equals the number of fixed elements in *B* by the permutation *g*∈*G*. Then the character associated to the induced representation *G *→* GL*(⊗^*n*^*W*) is *χ*^*n*^, the *n*-th power of *χ*.

We write *N*_1_,…,*N*_*t*_ for the irreducible representations of *G* and *ω*_1_,…,*ω*_*t*_ for the corresponding irreducible characters, where *N*_1 _and *ω*_1 _will denote the trivial representation and trivial character, respectively. Maschke’s Theorem applied to the action of *G* described in (3) states that there is a decomposition of ⊗^*s*^*W* into its isotypic components:

(4) $${⊗}^{s}W={⊕}_{i=1}^{t}({⊗}^{s}W)[\omega_{i}],$$

where each (⊗^*s*^*W*)*ω*_*i*_ is isomorphic to a number of copies of the irreducible representation *N*_*i*_ associated to *ω*_*i*_, $({⊗}^{s}W)[\omega_{i}]≅N_{i}⊗C^{m_{i}(s)}$, for some non-negative integer *m*_*i*_(*s*) called the *multiplicity of*⊗^*s*^*W relative to ω*_*i*_. The isotypic component of  associated to the trivial representation will be denoted by $L^{G}$ and it is composed of the *n*-tensors invariant under the action of *G* defined in (3). If  is the equivariant evolutionary model associated to *G*, $L^{G}$ will also be denoted as $L^{M}$. It is easy to prove that $CV_{T}^{M}\subset L^{G}$ (see Lemma 4.3 of [5]).

We recall that the set *Ω*_*G *_= {*ω*_*i*_}_*i *= 1,…,*t*_of irreducible characters of *G* forms an orthonormal basis of the space of characters relative to the inner product defined by

(5) $$\langle f,h\rangle :=\frac{1}{\left|G\right|}\underset{g\in G}{\sum }f(g)\bar{h(g)}\text{.}$$

We introduce the following notion.

**Definition 12.** An *n*-*word overB* is an ordered sequence X=x_1_x_2_…x_*n*_, where every letter is taken from the alphabet *B*. The set of *n*-words is equivalent to the cartesian power *B*^*n*^and will be denoted by .

Words will be denoted in typewritter uppercase font (like X) and their letters in lowercase (like X). Sometimes it will be convenient to identify the [*n*]-tensors of the form x_1_⊗…⊗x_*n*_ with the *n*-words X = x_1_…x_*n*_. Consequently, we will identify  with the natural basis of . Given , we will denote by {X}_*G *_= {*g*X ∣* g *∈* G*} the *G*-*orbit of*X. We associate a *G*-invariant tensor, *τ*{X}_*G*_, to each orbit {X}_*G*_: $\tau {\left\{X\right\}}_{G}:=\sum_{g\in G}gX$. It is straightforward to see that every *G*-invariant tensor can be written as a linear combination of the tensors *τ*{X}_*G*_, X∈B. On the other hand, the set of different *τ*{X}_*G *_’s is linearly independent, since the corresponding *G*-orbits {X}_*G*_ have non-overlapping composition of the elements of .

#### Mixtures for equivariant models

For each x ∈* B*, we write *S*_*G*_(x) for the *stabiliser of*X under the action of *G*, that is, *S*_*G*_(x) = {*g *∈* G*:*g*·x = x}.

**Proposition 13.** Let *G* be a subgroup of $S_{4}$ such that *S*_*G*_(x_0_) = {*e*} for some x_0_∈*B*. Then every tensor of type *τ*{X}_*G*_, X∈B, lies in the image of $\Psi_{T}^{M_{G}}$ for some tree topology *T*. In particular, $L^{G}\subset D_{M_{G}}$.

*Proof.* For any *G*-orbit {Y}_*G*_, Y∈B, write $\tau {\left\{Y\right\}}_{G}=y_{1}⊗\ldots ⊗Y_{n}+\underset{g\neq e}{\sum }g\cdot Y_{1}⊗\ldots ⊗g\cdot Y_{n}$. We will explicitly associate a tree topology and parameters (*Π*,**A**) to it so that the tensor *τ*{T}_*G*_is equal to $\Psi_{T}^{M_{G}}$. To this aim, we denote by *B*(Y) the set of letters appearing in Y. Then for every z∈*B*(Y), consider the set $L_{z}^{Y}=\{i\in [n]:Y_{i}=z\}$, so that $\cup_{z\in B(Y)}L_{z}^{Y}=[n]$.

We construct a tree *T* on the set of taxa [*n*] in the following way. We join each taxa in $L_{z}^{Y}$ to a common node *v*_z_ by an edge. Then each vertex *v*_z _is joined to the root of the tree (we call it *r*) by an edge that we denote as *e*(z) (see Figure 1). Now, in the edges joining any *v*_z_ with some leaf in $L_{z}^{Y}$, we consider the identity matrix, while the matrix in *e*(z) is defined by taking

$$A_{i,j}^{e(z)}=\left\{\begin{array}{ll} 1 & \;\;\text{if}\;(i,j)=(h\cdot x_{0},h\cdot z)\;\text{for some}\;h\in G,\\ 0 & \;\text{otherwise.} \end{array}\right)$$

Finally, if *c* is the cardinality of {*x*_0_}_*G*_, define the distribution at the root *Π*=(*Π*_A_,*Π*_C_,*Π*_G_,*Π*_T_) by

$$\Pi_{z}=\left\{\begin{array}{ll} \frac{1}{c} & \;\;\text{if}\;z\in {\left\{x_{0}\right\}}_{G},\\ 0 & \;\text{otherwise.} \end{array}\right)$$

It is straightforward to check that these matrices and the vector *Π *are *G*-equivariant, so $\left(\Pi ,A)\in {\text{Par}}_{M_{G}}(T\right)$. Now, from (2) and the definition of *Π*, we can write

$$\begin{array}{ll} p_{x_{1}\ldots x_{n}}=\underset{\begin{array}{c} g\in G\\ {\left\{x_{z}\right\}}_{z\in B(Y)}\subset B \end{array}}{\sum }P_{x_{1}\ldots x_{n}}(g,{\left\{x_{z}\right\}}_{z\in B(Y)}) & \; \end{array}$$

where

$$\begin{array}{ll} \;P_{x_{1}\ldots x_{n}}(g,{\left\{x_{z}\right\}}_{z\in B(Y)})=\Pi_{g\cdot x_{0}}\underset{z\in B(Y)}{\prod }\left(A_{g\cdot x_{0},x_{z}}^{e(z)}\underset{j\in L_{x_{z}}^{Y}}{\prod }\delta_{x_{z},x_{j}}\right) & \; \end{array}$$

(here *δ*_*a*,*b*_ stands for the Kronecker delta, i.e. *δ*_*a*,*a *_= 1, *δ*_*a*,*b *_= 0 if *a *≠* b*). Moreover, from the definition of the matrix *A*^*e*(^^z^^)^, we have

$$\;A_{g\cdot x_{0},x_{z}}^{e(z)}=\left\{\begin{array}{ll} 1 & \;\;\text{if}\;(g\cdot x_{0},x_{z})=(h\cdot x_{0},h\cdot z)\;\text{for some}\;h\in G,\\ 0 & \;\text{otherwise.} \end{array}\right)$$

The hypothesis *S*_*G*_(*x*_0_) = {*e*} ensures that (*g*·x_0_,x_z_) = (*h*·x_0_,*h*·z) if and only if *g *=* h*. From this, it becomes clear that $P_{x_{1}\ldots x_{n}}(g,{\left\{x_{z}\right\}}_{z\in B(Y)})=0$ unless 

1. x_z _=* g *· z, for z ∈* B*, and

2. for each $i\in L_{z}^{Y}$, x_*i*_is equal to x_z _=* g *· z,

in which case $P_{x_{1}\ldots x_{n}}(g,{\left\{x_{z}\right\}}_{z\in B(Y)})=\Pi_{g\cdot x_{0}}=\frac{1}{c}$. It follows that

$$p_{x_{1}\ldots x_{n}}=\left\{\begin{array}{ll} 1 & \;\;\text{if}\;x_{1}\ldots x_{n}\in {\left\{Y\right\}}_{G},\\ 0 & \;\text{otherwise,} \end{array}\right)$$

and $\Psi_{T}^{M}(\Pi ,A)=\tau {\left\{Y\right\}}_{G}$. Moreover, as the set of *τ*{Y}_*G*_, for Y∈B, generates the vector space $L^{G}$, the second claim follows. □

**Remark 14.** The above result is not true if the hypothesis *S*_*G*_(x_0_) = 〈*e*〉 is removed. For example, if *G* = 〈(ACGT),(AG)〉 (so that M=K80), then *S*_*G*_(A) =* S*_*G*_(G) = {*e*,(CT)} and *S*_*G*_(C) =* S*_*G*_(T) = {*e*,(AG)}. In that case, it can be shown that the *G*-orbit {ACGT}_*G*_is not in $\text{Im}\Psi_{T}^{\mathrm{K80}}$ for any tree topology *T* with 4 leaves.

Since the above condition on the group holds for $G=S_{4}$, *G *= 〈(AT)(CG)〉, and *G *= 〈(AG),(ACGT)〉, we deduce the following claim.

**Corollary 15.** If *G* corresponds to any of the equivariant models K81, SSM or GMM, we have $L^{G}\subset D_{M_{G}}$.

In phylogenetics, an *invariant of a phylogenetic treeT* is an equation satisfied by the expected distributions of patterns at the leaves of *T*, irrespectively of the continuous parameters of the model . In the algebraic geometry setting, these are the equations satisfied by all $p\in CV_{T}^{M}$. Invariants were introduced by Lake (see [27]) and Cavender and Felsenstein (see [28]). A *phylogenetic invariant ofT* is an invariant of *T*, which is not an invariant of all other phylogenetic trees (under the same model ). Equivalently, *f* is a phylogenetic invariant of $CV_{T}^{M}$ if it is an invariant of $CV_{T}^{M}$ and there exists a tree topology *T*^′^ such that *f* is not an invariant of $CV_{T^{\prime }}^{M}$. In principle, phylogenetic invariants can be used for tree topology reconstruction purposes.

**Remark 16.** (a) It can be seen that the condition of trivial stabiliser for some element of *B* given in Proposition 13 guarantees that all the irreducible representations of *G* will be present in the decomposition of *W* into its isotypic components. Then, by using the results of [6], it follows that the corresponding equivariant model will have no linear phylogenetic invariants. This fact was already known for the models in the above corollary: see [29] for the GMM, [21] for the SSM and [30] for the K81. Here we provided an alternative proof based on elementary tools of group theory.

(b) The models JC69 and K80 are known to have linear phylogenetic invariants, but these are the only linear invariants which do not define hyperplanes containing $L^{G}$, as can be deduced from [3,30]. In fact, for these two models, the claim of the corollary is still true as stated in the following theorem. Nevertheless, we have not been able to provide a unified proof of this fact because of the different properties of the corresponding groups. There is no description of the space of linear invariants for other equivariant models not listed in Example 4, so we cannot claim that the result below still holds.

**Theorem 17.***If*$M_{G}$*is one of the equivariant evolutionary models**JC69**,**K80**,**K81**,**SSM**, or**GMM**, then the space of phylogenetic mixtures*$D_{M_{G}}$*coincides with*$L^{G}$*, and*$D_{sM_{G}}$*equals*$L^{G}\cap H$*.*

This theorem allows to identify the set of all phylogenetic mixtures $D_{M_{G}}$ with $L^{G}$, which is a vector subspace of  whose linear equations are easy to describe. In other words, $L^{G}$ is the smallest linear space containing the data coming from any mixture of trees evolving under the model $M_{G}$. One can therefore use $L^{G}$ to select the most suitable model for the given data. This has been studied in [7].

*Proof of Theorem 17.* For equivariant models we have that $CV_{T}^{M_{G}}\subset L^{G}$ for any tree *T*. Hence, by Lemma 10 and the definition of $D_{M_{G}}$, $D_{M_{G}}$ is a vector subspace of $L^{G}$.

From Corollary 15, we infer the equality $L^{G}=D_{M_{G}}$ for the models K81, SSM and GMM. For the other two models, JC69 and K80, it remains to prove that there does not exist any hyperplane *Π* containing $D_{M_{G}}$ and not containing $L^{G}$. If such a hyperplane existed, then it would contain all the points of $CV_{T}^{M_{G}}$ for any tree topology *T*. It suffices to prove that for these models there are no homogeneous linear polynomials vanishing on all tree topologies, except for the linear equations vanishing on $L^{G}$. This has been seen in Remark 16(b).

The equality $D_{sM_{G}}=L^{G}\cap H$ follows immediately from Lemma 10 and the first assertion in the statement of this theorem. 

**Remark 18.** We are indebted to one of the referees of this paper for pointing out that the preceeding result, as well as the second part of Proposition 13, can also be inferred from Proposition 4.9 of [5]: under the assumption that the stabiliser of some state is trivial, Draisma and Kuttler show that the star tree is the smallest algebraic variety containing the tensors *τ*{X}_*G*_, for *pure* tensors X(that is, tensors of rank 1). It follows that the set of mixtures on the star tree equals the space $L^{G}$.

**Remark 19.** It is not difficult to check that for M=K81, SSM or GMM, $D_{M}$ coincides with the space of mixtures on the star tree (see also [26], where the same result is proven for a 2-state model). On the contrary, this is not true for JC69 and K80 models because in this case the star tree lies in a smaller linear space as a consequence of the existence of phylogenetic linear equations (see Remark 16(b)).

#### Equations for the space $L^{G}$

Our goal here is to compute the dimension of $L^{G}$ for the groups associated to the equivariant models listed in Definition 4, and to list a set of independent linear equations defining this space.

**Proposition 20.** Using the notations above, 

(i) $\text{dim}L^{\mathrm{SSM}}=2^{2n-1}$,

(ii) $\dim L^{\mathrm{K81}}=4^{n-1}$,

(iii) $\dim L^{\mathrm{K80}}=2^{2n-3}+2^{n-2}$, and

(iv) $\dim L^{\mathrm{JC69}}=\frac{2^{2n-3}+1}{3}+2^{n-2}$.

*Proof.* Let  be any equivariant model. By definition, we know that $L^{G}$ is the isotypic component of ⊗^*n*^*W* associated to the trivial representation (⊗^*n*^*W*)[*ω*_1_]. Since the dimension of the trivial representation is one, it follows that the dimension of $L^{M}$ is precisely the multiplicity *m*_1_(*n*), i.e. the number of times the trivial representation appears in the decomposition of ⊗^*n*^*W* into isotypic components. This multiplicity *m*_1_(*n*) equals (see (5))

$$\begin{array}{ll} \langle \chi^{n},\omega_{1}\rangle =\frac{1}{\left|G\right|}\underset{g\in G}{\sum }\chi^{n}(g)\omega_{1}(g). & \; \end{array}$$

The proof ends by grouping the elements of *G* in the conjugacy classes of *G* for SSM, K81, K80, or JC69. Recall that the conjugacy classes of a group *G* are the disjoint sets of the form *C*(*g*)={*h*^−1^*gh*:*h*∈*G*}. If *C*_1_,…,*C*_*s*_ are the conjugacy classes for *G*, write $C(G)=(|C_{1}|,\ldots ,|C_{s}|)$ for the *s*-tuple of their cardinalities, so that $\overset{s}{\underset{i=1}{\sum }}|C_{i}|=|G|$. Recall that *χ*^*n*^(*g*_1_) =* χ*^*n*^(*g*_2_) whenever *g*_1_ and *g*_2_ lie in the same conjugacy class, so we can represent *χ*^*n *^by an *s*-tuple $\chi_{C(G)}^{n}=(t_{1},\ldots ,t_{s})$, where *t*_*i *_=* χ*^*n*^(*g*) for any *g *∈* C*_*i*_. Thus, we have $m_{1}(n)=\frac{1}{\left|G\right|}\overset{s}{\underset{i=1}{\sum }}\chi^{n}(g_{i})|C_{i}|$, where *g*_*i*_ is any element in the conjugacy class *c*_*i*_. The result for M=SSM, K81, K80, or JC69 follows by applying the Table 1.

**Table 1:** Details of the conjugacy classes of some permutation groups needed in the proof of Proposition 20

$G\leq S_{4}$	M	**Representatives of conj. classes**	C(G)	$\chi_{C(G)}^{n}$
〈(AT)(CG)〉	SSM	{*e*,(AT)(CG)}	(1,1)	(4^*n*^,0)
〈(AC)(GT),(AG)(CT)〉	K81	{*e*,(AT)(CG),(AC)(GT),(AG)(CT)}	(1,1,1,1)	(4^*n*^,0,0,0)
〈(ACGT),(AG)〉	K80	{*e*,(AC)(GT),(AG)(CT),(ACGT),(AG)}	(1,2,1,2,2)	(4^*n*^,0,0,0,2^*n*^)
$S_{4}$	JC69	{*e*,(AC)(GT),(ACGT),(AG),(ACG)}	(1,3,6,6,8)	(4^*n*^,0,0,2^*n*^,1)

For each permutation group in the column on the left, the corresponding equivariant model and conjugacy classes are described. For each conjugacy class, we give a list of representatives, its cardinality and the value taken by the character *χ*^*n *^on it.

□

Our next goal is to provide a set of independent linear equations for $L^{G}$. Before stating the main result, let us introduce some useful notation.

**Notation 21.** We consider the following subsets of $B=B^{n}$:

$$\begin{array}{lll} B_{0} & =\;\;\{A\ldots A,C\ldots C,G\ldots G,T\ldots T\},\; & \;\\ B_{AC|GT}\;\; & =\;\;{\left\{A,C\right\}}^{n}\cup {\left\{G,T\right\}}^{n},\; & \;\\ B_{AG|CT}\;\; & =\;\;{\left\{A,G\right\}}^{n}\cup {\left\{C,T\right\}}^{n},\; & \;\\ B_{AT|CG}\;\; & =\;\;{\left\{A,T\right\}}^{n}\cup {\left\{C,G\right\}}^{n},\;\text{and}\; & \;\\ B_{2} & =\;\;B_{AC|GT}\cup B_{AG|CT}\cup B_{AT|CG}.\; & \; \end{array}$$

The set $B_{0}$ is composed of all *n*-words with only one letter and it is contained in $B_{AC|GT}$, $B_{AG|CT}$, and $B_{AT|CG}$. Similarly, $B_{2}$ is composed of all *n*-words with two letters at most. It is straightforward to check that $|B_{AC|GT}|=|B_{AG|CT}|=|B_{AT|CG}|=2^{n+1}$ and $|B_{2}|=3\cdot 2^{n+1}-8$.

We adopt multiplicative notation for *n*-words, for instance, we write C^*l *^for the word $\underset{l}{\underset{⎵}{C\ldots C}}$, and (A^*l*^)(G^*m*^)x_*l* + *m* + 1_…x_*n *_for $\underset{l}{\underset{⎵}{A\ldots A}}\underset{m}{\underset{⎵}{G\ldots G}}x_{l+m+1}\ldots x_{n}$, where x_*l* + *m* + 1_,…,x_*n *_represent any letters.

The main result of this section is the following:

**Theorem 22. ***A set of linearly independent equations*$E^{M}$*defining*$L^{M}$*for*= *JC69**,**K80**,**K81**, or **SSM **is given by*

$E^{\mathrm{SSM}}$ : equations *p*_X _=* p*_(__A__T__)(__C__G__)__X_for all X∈B with x_1 _∈ {A,C};

$E^{\mathrm{K81}}$ : the equations in $E^{\mathrm{SSM}}$, and the equations {*p*_X _=* p*_(__A__T__)(__C__G__)__X_} for all X∈B with x_1 _= A;

$E^{\mathrm{K80}}$ : the equations in $E^{\mathrm{K81}}$, plus the equations {*p*_X _=* p*_(__A__)(__G__)__X_} for all $X\in B\backslash B_{AC|GT}$ having x_1 _= A and satisfying the following condition: if T appears in X, then there is a C in a preceding position;

$E^{\mathrm{JC69}}$ : the equations in $E^{\mathrm{K80}}$, together with the equations {*p*_X _=* p*_(__A__T__)__X_} for all $X\in B_{AC|GT}\backslash B_{0}$ of the form (A^*l*^)(C^*m*^)x_*l* + *m* + 1_…x_*n*_; plus the equations {*p*_X _=* p*_(__A__C__)__X_} and {*p*_X _=* p*_(__A__T__)__X_} for all $X\in B\backslash B_{2}$ of the form (A^*l*^)(C^*m*^)x_*l* + *m* + 1_…x_*n *_and satisfying the condition: if Tappears in X, then there is a G in a preceding position.

The number of equations added in each case is 2^2*n*−1 ^for SSM, 2^2*n*−2^ for K81, 2^2*n*−3^−2^*n*−2^ for K80, and $2^{n-1}-1+2(\frac{2^{2n-3}+1}{3}-2^{n-2})$ for JC69.

Before proving this theorem, we explain how these sets of equations were obtained. Notice that a system of linear equations of $L^{G}$ is given by

$$\begin{array}{ll} \left\{p_{gX}=p_{X}|g\in G,\;X\in B\right\}. & \; \end{array}$$

The role played by the *G*-orbits on  becomes apparent. Indeed, the idea is to relate the equations to the orbits of a subgroup of *G*. To this aim, let *H* be a subgroup of *G* and write *H *\* G *= {*Hg*:*g *∈* G*} for the set of right cosets of *H* in *G*. We consider a transversal of *H *\* G*, i.e. a collection {*g*_1_,…,*g*_[*G*:*H*]_} such that $G={⊔}_{i=1}^{\left[G:H\right]}Hg_{i}$. Then the orbit of any X∈B can be decomposed as

(6) $${\left\{X\right\}}_{G}=\underset{i=1,\ldots ,[G:H]}{⋃}{\left\{g_{i}X\right\}}_{H}.$$

This decomposition establishes the connection between the *G*-orbits and the *H*-orbits. In order to obtain a system of equations for $L^{G}$, once $E^{H}$ has been computed, it is enough to add the equations involving the permutations in a transversal {*g*_1 _=* e*,*g*_2_,…,*g*_[*G*:*H*]_} of *H *\* G*:

$$\left(\begin{array}{l} p_{X}=p_{g_{2}X}\\ p_{X}=p_{g_{3}X}\\ \ldots \\ p_{X}=p_{g_{\left[G:H\right]}X} \end{array}\right\}\text{for all}\;X\in B.$$

Notice that the union in (6) is not necessarily disjoint as it may happen that {*g*_*i*_X}_*H *_= {*g*_*j*_X}_*H*_ for *i *≠* j*. In this case, the equality $p_{g_{j}X}=p_{g_{j}X}$ already holds in the space $L^{H}$ and does not provide any new restriction. In order to avoid this situation and obtain a minimal set of equations for $L^{G}$, we request the special conditions on the X∈B in the statement of the theorem.

*Proof.* For each model , we prove that the corresponding equations are linearly independent and there are as many equations as the codimension of $L^{M}$. By Proposition 20, the codimension of $L^{M}$ is 2^2*n*−1 ^for SSM, 3·4^*n*−1 ^for K81, 7·2^2*n*−3^−2^*n*−2 ^for K80, and $4^{n}-\frac{2^{2n-3}+1}{3}-2^{n-2}$ for JC69. In the sequel, we refer to the groups by the name of the equivariant model associated to them.

SSM: As SSM is the group {*e*,(AT)(CG)}, a set of equations for SSM is {*p*_X _=* p*_(__A__T__)(__C__G__)__X_}. Fixing x_1_in {A,C} we obtain 2^2*n*−1 ^linearly independent equations (equations involving different coordinates). The codimension of $L^{\mathrm{SSM}}$ is equal to 2^2*n*−1^, which coincides with the number of equations given, and thus this set of equations defines $L^{\mathrm{SSM}}$.

K81: Since a transversal of SSM\K81is {*e*,(AC)(GT)}, the hyperplanes *p*_X _=* p*_(__A__T__)(__C__G__)__X _contain $L^{\mathrm{K81}}$ but not $L^{\mathrm{SSM}}$. Moreover, using (6) we see that the orbit {X}_K81 _decomposes into the disjoint union of {X}_SSM _and {(AC)(GT)X}_SSM _for any X∈B. Therefore, the equations given for K81 involve different coordinates than those in $E^{\mathrm{SSM}}$. Requiring x_1 _= A, we obtain 4^*n*−1^linearly independent new equations. Thus $E^{\mathrm{K81}}$ defines the space $L^{\mathrm{K81}}$ because the number of linearly independent equations provided, 2^2*n*−1^ + 4^*n*−1 ^= 3·4^*n*−1^, coincides with the codimension of $L^{\mathrm{K81}}$.

K80: The set {*e*,(AG)} is a transversal of K81\K80. In order to show that the equations provided are linearly independent to those of $E^{\mathrm{K81}}$, we apply (6) to this transversal to obtain {X}_K80 _= {X}_K81_∪{(AG)X}_K81_. If $X\notin B_{AG|CT}$, then {(AG)X}_K81 _and {X}_K81_are disjoint, so each equation *p*_X _=* p*_(__A__)(__G__)__X _is linearly independent from $E^{\mathrm{K81}}$. The set $B\backslash B_{AG|CT}$ has cardinal 4^*n*^−2^*n* + 1^and, if $X\in B\backslash B_{AG|CT}$, each orbit {X}_K80 _has cardinality 8. Therefore, the number of different orbits for $X\in B\backslash B_{AG|CT}$ is (4^*n*^−2^*n* + 1^)/8 = 2^2*n*−3^−2^*n*−2^. Moreover, the choice of X’s in $B\backslash B_{AG|CT}$ with *x*_1 _= A and satisfying “if Tappears in X, there is a C in a preceding position” guarantees that we take only one element in each {X}_K80_, and thus we are adding exactly one equation for each of these X^′^*s*. Overall, there are 3·4^*n*−1^ + (2^2*n*−3^−2^*n*−2^) = 7·2^2*n*−3^−2^*n*−2 ^linearly independent equations in $E^{\mathrm{K80}}$. This number coincides with the codimension of $L^{\mathrm{K80}}$ and these equations define $L^{\mathrm{K80}}$.

JC69: A transversal of K80\JC69 is {*e*,(AC),(AT)}, therefore (6) applies to give ${\left\{X\right\}}_{\mathrm{JC69}}={\left\{X\right\}}_{\mathrm{K80}}\cup {\left\{(AC)X\right\}}_{\mathrm{K80}}\cup {\left\{(AT)X\right\}}_{\mathrm{K80}}$. Summing up, there are

$$7\cdot 2^{2n-3}-2^{n-2}+\left(2^{n-1}-1+2\left(\frac{1}{3}(2^{2n-3}+1)-2^{n-2}\right)\right)$$

linearly independent equations in $E^{\mathrm{JC69}}$ that contain $L^{\mathrm{JC69}}$. As this number is equal to the codimension $4^{n}-\frac{2^{2n-3}+1}{3}-2^{n-2}$ of $L^{\mathrm{JC69}}$, the proof is complete.

if $X\in B_{AC|GT}\backslash B_{0}$, then {(AC)X}_K80 _= {X}_K80 _and {X}_JC69 _is the disjoint union of {X}_K80_and {(AT)X}_K80_. As such, each equation *p*_X _=* p*_(__A__T__)__X_is linearly independent from $E^{\mathrm{K80}}$. Moreover, if $X\in B_{AC|GT}\backslash B_{0}$ is of the form (A^*l*^)(C^*m*^)x_*l* + *m* + 1_…x_*n*_, we have 2^*n*−1^−1 such equations and they are linearly independent.

if $X\in B\backslash B_{2}$ then the three orbits {(AC)X}_K80_, {(AT)X}_K80_, and {X}_K80 _have 8 elements each and are disjoint. Therefore, for these X’s, each equation of type {*p*_X _=* p*_(__A__C__)__X_} or {*p*_X _=* p*_(__A__T__)__X_} is linearly independent from $E^{\mathrm{K80}}.$ Moreover, as $B\backslash B_{2}$ has cardinal 4^*n*^−3·2^*n* + 1^ + 8 and is covered by these orbits, we have $\frac{4^{n}-3\cdot 2^{n+1}+8}{24}=\frac{1}{3}(2^{2n-3}+1)-2^{n-2}$ different orbits. The restriction to the elements of the form (A^*l*^)(C^*m*^)x_*l* + *m* + 1_…x_*n *_and satisfying that “if Tappears in X, there is some G in a preceding position” guarantees that the equations are written only only once for each orbit.

All the equalities among orbits used in this proof are summarized in Table 2.

**Table 2:** Equalities among orbits used in the proof of Theorem 22

	**{**X**}**_**GMM**_	**{**X**}**_**SSM**_	**{**X**}**_**K81**_	**{**X**}**_**K80**_	**{**X**}**_**JC69**_
$B_{0}$	{X}	⋯∪{(AT)(CG)X}	$\cdots \cup {\left\{(AC)(GT)X\right\}}_{\mathrm{SSM}}$	…	…
$B_{AG|CT}$	”	”	”	…	$\cdots \cup {\left\{(AC)\text{X}\right\}}_{\text{K80}}$
$B_{AC|GT}$	”	”	”	$\cdots \cup {\left\{(AG)X\right\}}_{\mathrm{K81}}$	$\cdots \cup {\left\{(AT)X\right\}}_{\mathrm{K80}}$
$B_{AT|CG}$	”	”	”	$\cdots \cup {\left\{(AG)X\right\}}_{\mathrm{K81}}$	$\cdots \cup {\left\{(AC)X\right\}}_{\mathrm{K80}}$
$B\backslash B_{2}$	”	”	”	$\cdots \cup {\left\{(AG)X\right\}}_{\mathrm{K81}}$	$\cdots \cup {\left\{(AC)X\right\}}_{\mathrm{K80}}\cup {\left\{(AT)X\right\}}_{\mathrm{K80}}$

For each *word*Xin , the orbit in the model that indexes the column is described. The dots  mean *the set on the left* and ” means *the set on the top*.

□

**Remark 23.** The sets of equations of Theorem 22 has been successfully used in [7] for model selection. Although the dimensions of these linear spaces are exponential in *n*, in practice it is not necessary to consider the full set of equations, but only those containing the patterns observed in the data. This is crucial for the applicability of the method, since the number of different columns in an alignment is really small compared to the dimension of these spaces.

**Example 24.** As an example, we compute a minimal system of equations for SSM, K81, K80, and JC69 in the case of 3 leaves.

*Equations for*$L^{\mathrm{SSM}}$: $E^{\mathrm{SSM}}$ is composed of the following equations:

$$\begin{array}{lll} & p_{AAA}=p_{TTT},\;\;p_{AAC}=p_{TTG},\;\;p_{AAG}=p_{TTC},\; & \;\\ & p_{AAT}=p_{TTA},\;\;p_{ACA}=p_{TGT},\;\;p_{ACC}=p_{TGG},\; & \;\\ & p_{ACG}=p_{TGC},\;\;p_{ACT}=p_{TGA},\;\;p_{AGA}=p_{TCT},\; & \;\\ & p_{AGC}=p_{TCG},\;\;p_{AGG}=p_{TCC},\;\;p_{AGT}=p_{TCA},\; & \;\\ & p_{ATA}=p_{TAT},\;\;p_{ATC}=p_{TAG},\;\;p_{ATG}=p_{TAC},\; & \;\\ & p_{ATT}=p_{TAA},\;\;p_{CAA}=p_{GTT},\;\;p_{CAC}=p_{GTG},\; & \;\\ & p_{CAG}=p_{GTC},\;\;p_{CAT}=p_{GTA},\;\;p_{CCA}=p_{GGT},\; & \;\\ & p_{CCC}=p_{GGG},\;\;p_{CCG}=p_{GGC},\;\;p_{CCT}=p_{GGA},\; & \;\\ & p_{CGA}=p_{GCT},\;\;p_{CGC}=p_{GCG},\;\;p_{CGG}=p_{GCC},\; & \;\\ & p_{CGT}=p_{GCA},\;\;p_{CTA}=p_{GAT},\;\;p_{CTC}=p_{GAG},\; & \;\\ & p_{CTG}=p_{GAC},\;\;p_{CTT}=p_{GAA}.\; & \; \end{array}$$

*Equations for*$L^{\mathrm{K81}}$: $E^{\mathrm{K81}}$ is formed by $E^{\mathrm{SSM}}$ and

$$\begin{array}{lll} & p_{AAA}=p_{CCC},\;\;p_{AAC}=p_{CCA},\;\;p_{AAG}=p_{CCT},\; & \;\\ & p_{AAT}=p_{CCG},\;\;p_{ACA}=p_{CAC},\;\;p_{ACC}=p_{CAA},\; & \;\\ & p_{ACG}=p_{CAT},\;\;p_{ACT}=p_{CAG},\;\;p_{AGA}=p_{CTC},\; & \;\\ & p_{AGC}=p_{CTA},\;\;p_{AGG}=p_{CTT},\;\;p_{AGT}=p_{CTG},\; & \;\\ & p_{ATA}=p_{CGC},\;\;p_{ATC}=p_{CGA},\;\;p_{ATG}=p_{CGT},\; & \;\\ & p_{ATT}=p_{CGG}.\; & \; \end{array}$$

*Equations for*$L^{\mathrm{K80}}$: $E^{\mathrm{K80}}$ is formed by $E^{\mathrm{K81}}$ and

$$\begin{array}{ll} p_{AAG}=p_{GAA},\;\;p_{ACG}=p_{GCA},\;\;p_{ACT}=p_{GCT}, & \;\\ p_{AGA}=p_{GAG},\;\;p_{AGC}=p_{GAC},\;\;p_{AGG}=p_{GAA}. & \; \end{array}$$

*Equations for*$L^{\mathrm{JC69}}$: $E^{\mathrm{JC69}}$ is formed by $E^{\mathrm{K80}}$ and

$$\begin{array}{lll} & p_{AAC}=p_{TTC},\;p_{ACA}=p_{TCT},\;p_{ACC}=p_{TCC},\; & \;\\ & p_{ACG}=p_{CAG},\;p_{ACG}=p_{TCG}.\; & \; \end{array}$$

### Identifiability of phylogenetic mixtures

In this section we study the identifiability of phylogenetic mixtures. To this end, we use projective algebraic varieties and techniques from algebraic geometry. It is not our intention to give the reader a background on these tools, so we refer to the algebraic geometry book [14] and, more specifically, to [10] for the usage of these techniques in the study of phylogenetic mixtures.

There is a natural isomorphism between the points lying in the hyperplane *H* considered above, $H=\{p=(p_{A\ldots A},\ldots ,p_{T\ldots T})\in L:\sum p_{x_{1}\ldots x_{n}}=1\}$, and the open affine subset $\left\{p=[p_{A\ldots A}:\cdots :p_{T\ldots T}]:\sum p_{x_{1}\ldots x_{n}}\neq 0\right\}$ of $P^{4^{n}-1}=P(L)$. We use the notation [*p*_A__…__A_:⋯:*p*_T__…__T_] for projective coordinates (in contrast to $\left(p_{\text{A}\ldots \text{A}},\ldots ,p_{\text{T}\ldots \text{T}}\right)$ used for affine coordinates). The *projective phylogenetic variety*$PV_{T}^{M}$ associated to a phylogenetic tree *T* is the projective closure in P(L) of the image of the stochastic parameterization $\phi_{T}^{M}$ defined above. That is, it is the smallest projective variety in P(L) containing $\text{Im}\;\phi_{T}^{M}$ via the above isomorphism.

In what follows, we explain the relationship between this new variety and $CV_{T}^{M}$ and $V_{T}^{M}$. By Remark 7, it becomes clear that $CV_{T}^{M}$ equals the affine cone over the projective phylogenetic variety $PV_{T}^{M}$ (for the general Markov model, see also [23], Proposition 1). This implies that $\dim CV_{T}^{M}=\dim PV_{T}^{M}+1$, and if $p=(p_{\text{A…A}},\ldots ,p_{\text{T…T}})$ belongs to $CV_{T}^{M}$, then *q *:=* p*_A__…__T_:⋯:*p*_T__…__T_ belongs to $PV_{T}^{M}$. Moreover, if $\lambda :=\sum p_{x_{1}\ldots x_{n}}$ is not zero, then $\left(\frac{p_{A\ldots A}}{\lambda },\ldots ,\frac{p_{T\ldots T}}{\lambda }\right)$ is a point in the affine stochastic phylogenetic variety $V_{T}^{M}$.

Before defining identifiability of mixtures, we consider the following construction of projective algebraic varieties.

**Definition 25.** Given two projective varieties $X,Y\subset P^{m}$, the *join of X and Y*, *X *∨* Y*, is the smallest variety in $P^{m}$ containing all lines $\bar{\mathrm{xy}}$ with *x *∈* X*, *y*∈*Y*, and *x *≠* y*(see [14], 8.1 for details). Similarly, one defines the *join of projective varieties*$X_{1},\ldots ,X_{h}\subset P^{m}$, ∨*i *= 1*hX*_*i*_, as the smallest subvariety in $P^{m}$ containing all the linear varieties spanned by $x_{1},\ldots ,x_{h}$ with *x*_*i *_∈* X*_*i *_and *x*_*i *_≠* x*_*j*_. It is known that

$$\begin{array}{ll} \dim(\vee_{i=1}^{h}X_{i})\leq \min\{\overset{h}{\underset{i=1}{\sum }}\dim(X_{i})+h-1,m\}. & \; \end{array}$$

The right hand side of this inequality is usually known as the *expected dimension* of ∨*i *= 1*hX*_*i*_.

For instance, if we consider the join $\vee_{i=1}^{h}PV_{T_{i}}^{M}$ for certain tree topologies *T*_*i *_on the leaf set [*n*] and a given evolutionary model , then there is a (dominant rational) map

(7) $$\;PV_{T_{1}}^{M}\times PV_{T_{2}}^{M}\times \ldots \times PV_{T_{h}}^{M}\times P^{h-1}--\to \vee_{i=1}^{h}PV_{T_{i}}^{M}\subset P(L),$$

which is the projective closure of the parameterization $\phi_{T_{1}}\vee \ldots \vee \phi_{T_{h}}$ defined by

$$\begin{array}{lll} \;\mathrm{Pa}r_{sM}(T_{1})\times \ldots \times \mathrm{Pa}r_{sM}(T_{h})\times \Omega & \to & L\\ \;\left(\xi_{1},\ldots ,\xi_{h},a\right) & \mapsto & \underset{j}{\sum }a_{i}\phi_{T_{i}}^{M}(\xi_{i}). \end{array}$$

Here, $\Omega =\{a=(a_{1},\ldots ,a_{h})|\underset{i}{\sum }a_{i}=1\}$ is isomorphic to an affine open subset of $P^{h-1}$. In this setting, an *h*-mixture on {*T*_1_,…,*T*_*h*_} corresponds to a point in the variety $\vee_{i=1}^{h}PV_{T_{i}}^{M}$. We will use this algebraic variety to study the identifiability of phylogenetic mixtures.

When considering unmixed models  on trivalent trees on *n* taxa, *generic identifiability of the tree topology* is equivalent to the projective varieties $PV_{T}^{M}$ and $PV_{T^{\prime }}^{M}$ being different when *T *≠* T*^′^(see [31]). The identifiability of the continuous parameters must take into account the possibility of permuting the labels of the states at the interior nodes, as such permutations give rise to the same joint distribution at the leaves. In the language of algebraic geometry, *generic identifiability of the continuous parameters* of the model implies that the map $\phi_{T}^{M}$ is generically finite (i.e. the preimage of a generic point is a finite number of points; see [31]). In this case, the fiber dimension Theorem ([14], Theorem 11.12) applies and we have that $\dim PV_{T}^{M}$ is equal to the number of stochastic parameters of the model, $\dim\mathrm{Pa}r_{sM}(T)$. Therefore, if the continuous parameters are generically identifiable for the unmixed trees under , then the dimension of the variety $PV_{T}^{M}$ is the same for all trivalent tree topologies on *n* taxa. This dimension is denoted by $s_{M}(n)$.

**Example 26.** The tree topologies and the continuous parameters are generically identifiable for the unmixed equivariant models JC69, K80, K81, SSM, and GMM on trees with any number of leaves (see [9] and [6], Corollary 3.9).

From now on we only consider trees without nodes of degree 2, so that the number of free stochastic parameters on a phylogenetic tree on *n* taxa under  is $\leq s_{M}(n)$.

We recall the definition of generic identifiability of the tree topologies on *h*-mixtures (see [10]).

**Definition 27.** The *tree topologies* on *h*-mixtures under  are *generically identifiable* if for any set of trivalent tree topologies {*T*_1_,…,*T*_*h*_} and a generic choice of $(\xi_{1},\ldots \xi_{h},a)\in \mathrm{Pa}r_{sM}(T_{1})\times \ldots \times \mathrm{Pa}r_{sM}(T_{h})\times \Omega$, the equality

$$\;\phi_{T_{1}}\vee \ldots \vee \phi_{T_{h}}(\xi_{1},\ldots \xi_{h},a)=\phi_{T_{1}^{\prime }}\vee \ldots \vee \phi_{T_{h}^{\prime }}(\xi_{1}^{\prime },\ldots \xi_{h}^{\prime },a^{\prime }),$$

for tree topologies $\left\{T_{1}^{\prime },\ldots ,T_{h}^{\prime }\right\}$ and $(\xi_{1}^{\prime },\ldots \xi_{h}^{\prime },a^{\prime })\in \mathrm{Pa}r_{sM}(T_{1}^{\prime })\times \ldots \times \mathrm{Pa}r_{sM}(T_{h}^{\prime })\times \Omega$ implies

$$\{T_{1},\ldots ,T_{h}\}=\{T_{1}{,}^{\prime }\ldots ,T_{h}^{\prime }\}.$$

In terms of algebraic varieties this is equivalent to saying that the variety $\vee_{i=1}^{h}PV_{T_{i}}^{M}$ is not contained in $\vee_{i=1}^{h}PV_{T_{i}^{\prime }}^{M}$ and vice versa.

The tree topologies are the discrete parameters of *h*-mixtures. When considering the continuous parameters of *h*-mixtures, the above mentioned label-swapping can be disregarded. We give the following definition according to [12].

**Definition 28.** The *continuous parameters* of *h*-mixtures on $T_{1},\ldots ,T_{h}$ under an evolutionary model  are *generically identifiable* if, for a generic choice of stochastic parameters $\left(\xi_{1},\ldots ,\xi_{h},a\right)$, the equality

$$\phi_{T_{1}}\vee \ldots \vee \phi_{T_{h}}(\xi_{1},\ldots \xi_{h},a)=\phi_{T_{1}}\vee \ldots \vee \phi_{T_{h}}(\xi_{1}^{\prime },\ldots \xi_{h}^{\prime },a^{\prime })$$

for stochastic parameters $\left(\xi_{1}^{\prime },\ldots ,\xi_{h}^{\prime },a^{\prime }\right)$ implies that there is a permutation $\sigma \in S_{h}$ such that $\sigma \cdot (T_{1},\ldots ,T_{h})=(T_{1},\ldots ,T_{h})$, $\xi_{i}^{\prime }=\xi_{\sigma (i)}$, and $a_{i}^{\prime }=a_{\sigma (i)}$ for i=1,…r. In other words, we only allow swapping of the continuous parameters when at least two tree topologies coincide.

**Definition 29.** An *h*-mixture under a model  is said to be *identifiable* if both its tree topologies and its continuous parameters are generically identifiable.

In terms of algebraic varieties, generic identifiability of continuous parameters on *h*-mixtures implies that the generic fibers (i.e. preimages of generic points) of the map $\phi_{T_{1}}\vee \ldots \vee \phi_{T_{h}}$ are finite. In this case, the fiber dimension theorem applied to (7) (cf. [14], Theorem 11.12) gives

$$\begin{array}{ll} \dim(\vee_{i=1}^{h}PV_{T_{i}})=\overset{h}{\underset{i=1}{\sum }}\dim(PV_{T_{i}})+h-1. & \; \end{array}$$

The following result demonstrates the need for careful inspection of identifiability of mixtures with many components (i.e. large values of *h*).

**Theorem 30.**Let [*n*] be a set of taxa and  be an evolutionary model for which the continuous parameters are generically identifiable on trivalent (unmixed) trees. In addition, let $s_{M}(n)$ be the dimension of $PV_{T}^{M}$ for any trivalent tree *T*, and set $h_{0}(n):=\frac{\dim D_{M}}{s_{M}(n)+1}$. Then the *h*-mixtures of trees on [*n*] evolving under  are not identifiable for *h *≥* h*_0_(*n*).

**Remark 31.** Note that, in the above definition of *h*_0_(*n*), $\dim D_{M}$ also depends on *n*.

**Corollary 32.** Let [*n*] be a set of taxa and  be one of the equivariant models JC69, K80, K81, SSM, or GMM. Then the phylogenetic *h*-mixtures under these models are not identifiable for *h *≥* h*_0_(*n*), where

$$h_{0}\left(n\right)=\left\{\begin{array}{l} \frac{4^{n}}{12(2n-3)+4},\;\text{if}\;M=\text{GMM},\\ \frac{2^{2n-1}}{6(2n-3)+2},\;\text{if}\;M=\text{SSM},\\ \frac{4^{n-1}}{3(2n-3)+1},\;\text{if}\;M=\text{K81},\\ \frac{2^{2n-3}+2^{n-2}}{2(2n-3)+1},\;\text{if}\;M=\text{K80},\\ \frac{2^{2n-3}+3\cdot 2^{n-2}+1}{3(2n-2)},\;\text{if}\;M=\text{JC69}. \end{array}\right)$$

*Proof.* Theorem 17 shows that $L^{M}=D_{M}$ and Proposition 20 gives the dimension of $L^{M}$ in each case. Next, we calculate: *s*_GMM_(*n*) = 12(2*n*−3) + 3, *s*_SSM_(*n*) = 6(2*n*−3) + 1, *s*_K81_(*n*) = 3(2*n*−3), *s*_K80_(*n*) = 2(2*n*−3), and *s*_JC69_(*n*) = 2*n*−3. Applying Theorem 30, we conclude the proof. □

**Example 33.** Consider the Kimura 3-parameter model K81 on *n *= 4 taxa. For any *h *≥ 4, phylogenetic *h*-mixtures are not identifiable by Corollary 32. We are not aware of any result proving that mixtures of 2 or 3 different tree topologies under this model are identifiable (either for the tree parameters or for the continuous parameters).

**Example 34.** Consider the Jukes-Cantor model JC69 on *n *= 4 taxa. Then Corollary 32 tells us that for *h *≥ 3, *h*-mixtures are not identifiable. Therefore, for this particular model on four taxa the cases in which the identifiability holds are known: the tree and the continuous parameters are generically identifiable for the unmixed model; the tree parameters are generically identifiable for 2-mixtures ([10], Theorem 10); the continuous parameters are generically identifiable for 2-mixtures on different tree topologies and not identifiable for the same tree topology ([10], Theorem 23); neither the continuous parameters nor the tree topologies are generically identifiable for mixtures with more than two components (Corollary 32).

*Proof of Theorem 30.* Let $\mathrm{edim}(h):=hs_{M}(n)+h-1$. Then the variety $\vee_{i=1}^{h}PV_{T_{i}}$ has dimension ≤* edim*(*h*). Indeed, as $\vee_{i}\phi_{T_{i}}$ is a parameterization of an open subset of $\vee_{i=1}^{h}PV_{T_{i}}$, then the dimension of $\vee_{i=1}^{h}PV_{T_{i}}$ is less than or equal to $\sum \dim PV_{T_{i}}+h-1$. Moreover, the dimension of $PV_{T_{i}}$ is equal to $s_{M}(n)$ if *T*_*i*_ is trivalent (since the continuous parameters for the unmixed models under consideration are generically identifiable) and is less than $s_{M}(n)$ for non-trivalent trees. Therefore, $\dim(\vee_{i=1}^{h}PV_{T_{i}})\leq \mathrm{edim}(h)$.

If all *T*_*i*_ are trivalent trees, then $\sum \dim PV_{T_{i}}+h-1=\mathrm{edim}(h)$ and, therefore, $\dim(\vee_{i=1}^{h}PV_{T_{i}})<\mathrm{edim}(h)$ if and only if $\dim(\vee_{i=1}^{h}PV_{T_{i}})<\sum \dim PV_{T_{i}}+h-1$. Moreover, by the fiber dimension theorem applied to $\vee \phi_{T_{i}}$, the equality of dimensions holds if and only if the generic fiber of $\vee \phi_{T_{i}}$ has dimension 0. In particular, if $\dim(\vee_{i=1}^{h}PV_{T_{i}})<\mathrm{edim}(h)$, then the continuous parameters of this phylogenetic mixture are not identifiable.

As $h_{0}(n)=\frac{\dim D_{M}}{s_{M}(n)+1}$, we have that $\mathrm{edim}(h_{0}(n))=h_{0}(n)(s_{M}(n)+1)-1=\dim D_{M}-1$. Now, we fix an h∈N with *h *≥* h*_0_(*n*), so that $\mathrm{edim}(h)\geq \dim(D_{M})-1$.

There are two possible scenarios: 

(a) For any set of tree topologies $\left\{T_{1},\ldots ,T_{h}\right\}$, the dimension of $\vee_{i=1}^{h}PV_{T_{i}}$ is less than $\dim(D_{M})-1$.

(b) There exists a set of tree topologies $\left\{T_{1},\ldots ,T_{h}\right\}$ for which $\text{dim}(\vee_{i=1}^{h}PV_{T_{i}})=\text{dim}(D_{M})-1.$

Case (a) implies that the dimension of $\vee_{i=1}^{h}PV_{T_{i}}$ is less than *edim*(*h*) for any set of trivalent tree topologies $\left\{T_{1},\ldots ,T_{h}\right\}$. Based on the conclusions drawn above, this implies that the continuous parameters are not generically identifiable.

In case (b), $\vee_{i=1}^{h}PV_{T_{i}}$ coincides with $P(D_{M})$. Indeed, $\vee_{i=1}^{h}PV_{T_{i}}$ is contained in $P(D_{M})$, both varieties are irreducible, and $\text{dim}(\vee_{i=1}^{h}PV_{T_{i}})=\text{dim}(D_{M})-1=\text{dim}(P(D_{M}))$, which implies that both varieties coincide. In particular, any *h*-mixture (which is a point in $P(D_{M})$) would be contained in $\vee_{i=1}^{h}PV_{T_{i}}$, and therefore the topologies are not generically identifiable. 

**Remark 35.** The negative result of Theorem 30 should be complemented with the following positive result of Rhodes and Sullivant in [12]: if >=GMM and one restricts to *h*-mixtures on the same trivalent tree topology *T*, then the tree topology and the continuous parameters are generically identifiable if $h<4^{\lceil \frac{n}{4}\rceil -1}$.

## Conclusions

In this paper, we have dealt with the space of phylogenetic mixtures for evolutionary equivariant models. We have shown that for the case of the Jukes-Cantor model, the Kimura models with two or three parameters, the strand symmetric model and the general Markov model, this linear space is defined by the set of linear equations satisfied by the distributions of the patterns at the leaves of a tree that evolves under that model. It follows that this space completely characterizes the model. The use of tools from group theory and group representation theory played a major role, and allowed us to design a procedure to produce minimal systems of equations for these spaces and for any number of taxa. This procedure has been implemented successfully in a new method for model selection in phylogenetics based on linear invariants (see [7]), which is available online at http://genome.crg.es/cgi-bin/phylo_mod_sel/AlgModelSelection.pl,.

In the last part of the paper, we proved new results concerning the identifiability of phylogenetic mixtures. Namely, we provided an upper bound for the number of components (classes) of a mixture so that the identifiability of both the continuous and the discrete parameters is still possible.

## Competing interests

The authors declare that they have no competing interests.

## Authors’ contributions

All authors contributed equally and the author names order is alphabetical. All authors read and approved the final manuscript.
